# Buildings Contemplated

**Published:** 1913-12-06

**Authors:** 


### Buildings Contemplated.

Bridlington.—The Local Government Board has held an inquiry into an application of the Town Council for permission to borrow £1,500 for the extension of the isolation hospital in accordance with plans prepared by Mr. E. R. Matthews, the borough surveyor.

Chester.—The City Corporation has decided to erect a tuberculosis pavilion adjoining the isolation hospital at Chester. Tenders for the work, in accordance with plans and specifications, which may be seen at the City Surveyor's Office, are invited, and it is hoped that the building will shortly be proceeded with.

Colney Hatch.—It is proposed, at a cost of £2,750, to make a number of structural alterations at Colney Hatch Mental Hospital, so as to provide improved accommodation for the nursing staff. The convalescent home is to be enlarged, and devoted entirely to the use of the nurses, while the cubicles at present used by the staff are to be adapted for the accommodation of patients.

